# A hitchhiker’s guide to autophagy

**DOI:** 10.1038/s44318-024-00160-y

**Published:** 2024-07-04

**Authors:** Susanna Tulli, Sascha Martens

**Affiliations:** 1 Max Perutz Labs, Vienna Biocenter Campus (VBC), Dr.-Bohr-Gasse 9, 1030 Vienna, Austria; 2 Max Perutz Labs, Department of Biochemistry and Cell Biology, University of Vienna, Dr.-Bohr-Gasse 9, 1030 Vienna, Austria

**Keywords:** Autophagy & Cell Death

## Abstract

A study identifies a new mechanism to specifically attach cytoplasmic components to lipidated ATG8 proteins during starvation-induced autophagy.

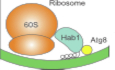

## Teaser

Macroautophagy (autophagy) mediates the degradation of cytoplasmic materials within lysosomes. In this issue, Takeda et al, identify an as-yet unknown mechanism that specifically attaches cytoplasmic components to lipidated ATG8 proteins during starvation-induced autophagy. This mechanism may be widely employed in autophagic pathways.

Autophagy is a conserved lysosomal degradation pathway in which cytoplasmic material is sequestered within double-membrane vesicles called autophagosomes. Autophagosomes are formed de novo and are first observed as small, flattened cisternae referred to as phagophores (or isolation membranes). The phagophores gradually surround cytoplasmic material as they grow. After their closure is complete, the autophagosomes fuse with the endolysosomal system or the vacuole in yeast and plants, where the material they contain is degraded (Chang et al, 2021).

Autophagy can be induced by various stimuli, the nature of which determines the degree of cargo selectivity (Zaffagnini and Martens, 2016). During starvation-induced autophagy, autophagosomes are thought to trap cytoplasmic content that is in the vicinity of the growing phagophore in a largely non-selective manner. Starvation-induced autophagy is therefore also often termed “bulk autophagy”. Alternatively, autophagy can also be induced by the appearance of harmful material such as damaged mitochondria, protein aggregates or pathogens. In these pathways, collectively referred to as cargo-induced autophagy, the autophagy machinery is recruited to the cargo material via cargo receptors and scaffold proteins. In *Saccharomyces cerevisiae*, this process is dependent on the scaffold protein Atg11. Later during the process, specific cargo is tethered to the nascent autophagosomal membrane by dedicated cargo receptors, which simultaneously bind the cargo and ATG8 family proteins which decorate the growing membrane (Rogov et al, 2014). Cargo receptors use Atg8 interacting motifs (AIMs, also called LC3-interacting regions (LIRs)) to bind ATG8 proteins (Noda et al, 2008; Pankiv et al, 2007). Notably, ATG8 family proteins are attached to the autophagosomal membrane in both bulk and cargo-induced autophagy.

Takeda et al (2024) describe a mechanism that confers specificity to starvation-induced bulk autophagy. This process tethers cargo with high specificity to the autophagosomal membrane but does not engage Atg11, a scaffold that is required for autophagosome biogenesis. For this reason, it hitchhikes with starvation-induced autophagy to deliver its cargo into the vacuole.

To investigate this process, Takeda and colleagues employed a method they previously developed (Kawamata et al, 2022) to analyze autophagosomal contents after nitrogen starvation or rapamycin treatment. Together with the expected autophagy-related proteins, they found enrichment of a small uncharacterized protein that they named Hab1 (highly enriched in autophagic bodies, previously annotated as Ybr285w). When compared to other cytosolic proteins, Hab1 was preferentially delivered to the vacuole. Interestingly, this process was dependent on the core autophagy machinery, but not on Atg11. Further analyses revealed that an amphipathic helix with membrane-binding properties at the N-terminus of Hab1 was essential for its delivery to the vacuole.

Consistent with the potential role of Hab1 as an autophagy receptor the authors identified and validated a functional AIM motif within the N-terminal part of the Hab1 protein, in direct proximity to the amphipathic helix. The authors therefore speculate that this would allow Hab1 to preferentially bind to lipidated and membrane-localized Atg8. Indeed, in co-immunoprecipitation experiments Hab1 showed a high preference for interaction with the lipidated form of Atg8.

As no major difference in autophagy flux was observed in Hab1-deficient cells, the authors conclude that Hab1 might not be involve in modulation of autophagosome biogenesis per se but rather act as an autophagy receptor. Interestingly, the degradation of Hab1 was dependent on Atg24 which was previously shown to be required for the sequestration of large cargo by autophagosomes (Kotani et al, 2023). Given that the N-terminus of Hab1 was already involved in membrane binding, Takeda and colleagues proceeded with cargo identification via immunoprecipitation using the Hab1 C-terminus followed by mass spectrometry analysis. Among the co-precipitating proteins ribosomal proteins were highly enriched. The interaction of Hab1 with ribosomes was confirmed by co-sedimentation experiments. Next, the authors examined how depletion and overexpression of Hab1 affected ribosomal turnover via starvation-induced autophagy. Only a minor reduction in the amount of ribosomal proteins was observed in autophagic bodies of cells lacking Hab1 but its overexpression led to an increase in the autophagic degradation of 60 S ribosomal subunits as well as 40 S subunits. Overexpressing Hab1 increased the density of ribosomes that were close to the inner autophagosomal membrane, while its depletion had the opposite effect. This indicated that, despite being a Hab1 cargo, turnover of ribosomes during autophagy occurs mostly via non-selective autophagy. A similar phenotype was previously described by Kraft et al (2008), who reported an increased turnover of a subset of ribosomal proteins during starvation with a preference for subunits of the 60 S subunit.

The capacity of Hab1 to tether its cargo to autophagosomal membranes by simultaneously binding the membrane and lipidated Atg8 is unprecedented. To test if this domain was sufficient to selectively degrade other cargos, the authors tethered the N-terminus of Hab1 to cytosolic Pho8∆60, mitochondria, and peroxisomes. In all three cases, the cargo was preferentially degraded by autophagy upon starvation. In addition, when mitophagy was compromised by the deletion of ATG11 and ATG32, the recruitment of Hab1 N-terminus to Tom70 was sufficient to restore it. These results show that this domain is sufficient to mediate the preferential degradation of various cellular structures by starvation-induced autophagy.

**Figure 1:**
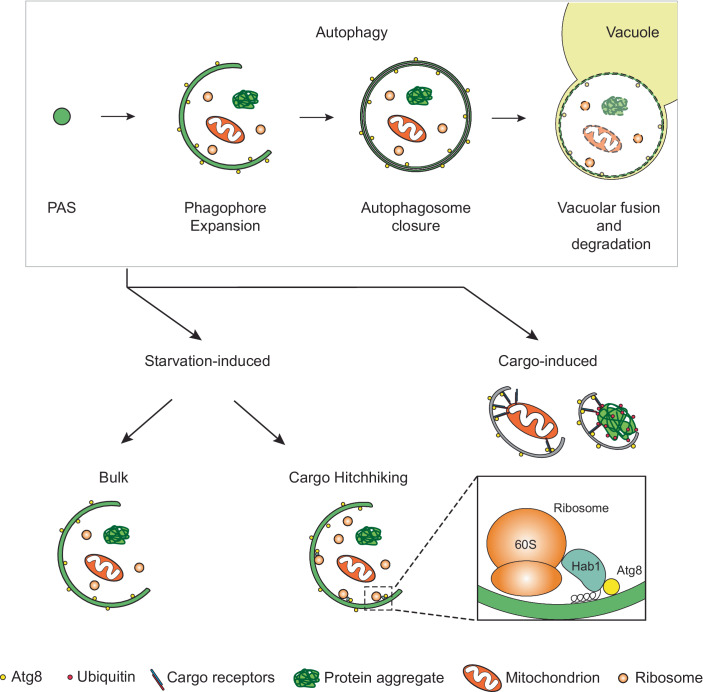
Scheme illustrating the varying degree of selectivity in different autophagy pathways. (Upper panel) Autophagosome biogenesis starting from initiation at the PAS (pre-autophagosomal structure), through phagophore expansion, to autophagosome fusion with the vacuole. (Lower panel) Autophagosome biogenesis can be induced by starvation as well as by the presence of specific cargo, such as damaged mitochondria or protein aggregates. Classically, starvation-induced autophagy was considered non-selective. However, Takeda et al (2024) show that ribosomes can be enriched as cargo in bulk autophagy by hitchhiking the Atg8-decorated membrane via Hab1.

Takeda et al (2024) describe a new way for the specific degradation of cargo material by non-selective bulk autophagy. Fittingly, the authors define this new mechanism as “cargo hitchhiking” as it exploits autophagosome biogenesis induced upon starvation to degrade a specific cargo (Fig. 1). The study raises several interesting questions. First, does Hab1 target a specific subset of ribosomes? Second, are there other proteins with similar properties in yeast and in complex eukaryotes? Proteomics approaches such as those employed by the authors in combination with bioinformatic screening for proteins with AIMs in proximity to amphipathic helices may identify further proteins with Hab1-like features. Notably, the preferential degradation of certain proteins, even by so-called starvation-induced bulk autophagy, has been long observed. Cargo receptors, including p62, are frequently used to assess autophagic flux as they are degraded very quickly. In these instances, the preference for the binding to the nascent autophagosomal membrane may be mediated by multimerization, which clusters the binding sites for the ATG8 family proteins conferring high avidity binding to ATG8-decorated membranes (Wurzer et al, 2015). Furthermore, it has recently been reported that in mammalian cells membrane protein cargoes are preferentially turned over by autophagy (Hickey et al, 2023). Are either of these observations also relevant to Hab1?

In summary, the fascinating mechanism identified by Takeda and colleagues regarding hitchhiking on starvation-induced autophagy for the degradation of selected material provides an important contribution to our understanding of the specificity of the autophagic process. Ultimately, these insights are crucial for our appreciation of the physiological roles of autophagy in various stress conditions.
